# Effects of bronchoscopic alveolar lavage–assisted mechanical ventilation on postoperative pulmonary infection and inflammatory factors in patients undergoing lung cancer surgery

**DOI:** 10.20452/wiitm.2024.17887

**Published:** 2024-07-23

**Authors:** Hui Zhu, Haiyang Lu

**Affiliations:** Department of Respiratory Endoscopy Center, West China Hospital, Sichuan University, Chengdu, Sichuan Province, China

**Keywords:** bronchoscopic alveolar lavage, mechanical ventilation, lung cancer, pulmonary infection

## Abstract

**INTRODUCTION:**

Patients undergoing lung cancer (LC) surgery often develop pulmonary infection (PI) due to poor sputum excretion ability leading to accumulation of secretions. Bronchoalveolar lavage (BAL) during fiberoptic bronchoscopy can be used to clear respiratory secretions and serve as a vehicle to locally deliver antibiotics that fight infection.

**AIM:**

This study explored the clinical efficacy of BAL‑assisted mechanical ventilation (MV) in patients with postoperative PI and its influence on inflammatory parameters.

**MATERIALS AND METHODS:**

A total of 90 LC patients with postoperative PI were enrolled and divided into an MV group (n = 45) and a BAL‑assisted MV group (n = 45). Therapeutic effects, respiratory mechanics, lung function parameters, blood gas indices, and levels of inflammatory parameters in peripheral blood were compared between the groups.

**RESULTS:**

In comparison with the MV group, the PI control time, MV duration, temperature recovery time, and Clinical Pulmonary Infection Score of patients in the BAL‑assisted MV group were greatly reduced. In both groups, airway resistance, peak airway pressure, and work of breathing decreased, while lung compliance increased. Maximum minute ventilation, total lung capacity, forced expiratory volume in 1 second, and maximum mid‑expiratory flow were substantially enhanced following each treatment. In addition, post‑treatment arterial oxygen pressure and saturation, pH value, and the ratio of partial pressure of oxygen in arterial blood to the fraction of inspiratory oxygen concentration increased, while partial pressure of carbon dioxide decreased in both groups. The levels of inflammatory markers (high‑sensitivity C‑reactive protein, procalcitonin, tumor necrosis factor α, high‑mobility group box 1, interleukin 6, and macrophage inflammatory protein 1α) in peripheral blood were decreased regardless of the treatment method (all *P* <0.05). All these findings were more pronounced in the BAL‑assisted MV group. Clinical effective rate in the BAL‑assisted MV group was higher than in the MV group (93.33% vs 77.78%; *P* <0.05).

**CONCLUSIONS:**

BAL‑assisted MV can achieve better results in treating postoperative PI than MV alone, effectively improving respiratory function and reducing systemic inflammatory response.

## INTRODUCTION

Lung cancer (LC) is a malignant tumor frequently observed in clinical practice. It is associated with high mortality and morbidity.^1^ Surgery is a common treatment for LC; however, it significantly impairs the immune function of patients and their ability to expel phlegm from the airways. Accumulation of lung secretions may lead to rapid growth and reproduction of pathogenic bacteria, and, finally, to pulmonary infection (PI) development.^2^^;^^3^ PI after LC increases surgical trauma of patients, which, in turn, prolongs infection, leading to a vicious cycle.^4^ PI is a common critical disease in respiratory medicine, mainly manifesting as excessive sputum volume, asthma, etc. If not controlled in time, it may cause multiple organ failure or even death.^5^ With the development of fiberscopy technology, bronchoalveolar lavage (BAL) performed during fiberoptic bronchoscopy can allow for injection of antibiotics into infection foci, thus playing a direct anti‑infection role. It can also effectively clear inflammatory secretions from the airways and improve the airway ventilation function.^6^^;^^7^ BAL has become an effective method for diagnosis and treatment of severe pneumonia.^8^ It consists in clearing the interior of the alveoli through perfusion of saline or other solutions. Combined with mechanical ventilation (MV), it can reduce the accumulation of pathogens and inflammatory mediators, thus reducing the incidence of infection and inflammation and the risk of postoperative complications.^9^^;^^10^ However, the actual effects of BAL‑assisted MV on postoperative infection and levels of inflammatory markers in patients undergoing LC surgery have not been systematically and comprehensively studied.

## AIM

This study aimed to evaluate the effects of BAL‑assisted MV on postoperative PI and levels of inflammatory parameters in patients undergoing LC surgery. It provides new ideas and strategies for improving the treatment of postoperative patients and sheds light on the mechanism of inflammation and infection after LC surgery.

## MATERIALS AND METHODS

### Materials

We en‑ rolled patients treated at the West China Hospital, Sichuan University between March 2021 and March 2023 who underwent LC surgery complicated with postoperative PI. The inclusion criteria comprised 1) LC confirmed by fiberoptic bronchoscopy and punitive biopsy, removed us‑ ing a minimally invasive technique (including thoracoscopic surgery or minimally invasive lobectomy); 2) confirmed postoperative PI meeting the diagnostic criteria outlined in the Guidelines for Diagnosis and Treatment of Hospital‑Acquired Pneumonia ^11^; 3) age of 18 to 75 years; and 4) indications for MV therapy. The patients were excluded if they met any the following criteria: 1) pulmonary tuberculosis, pulmonary fibrosis, or atelectasis; 2) preoperative PI; 3) other concomitant infectious diseases; 4) LC complicated by other malignant tumors, immune deficiency, or mental disorders; 5) severe liver and / or kidney dysfunction; 6) a recent history of immunosuppressant use; and 7) missing clinical data.

A total of 90 patients were eventually enrolled and randomly divided into 2 groups (45 patients in each group), according to the treatment method (MV vs BAL‑assisted MV). In the MV group, 27 men and 18 women were included; their mean (SD) age was 61.9 (4.7) years (range, 50–75 years), and their mean (SD) body mass index (BMI) was 21.4 (2) kg/m^2^. In the BAL‑assisted MV group, there were 26 men and 19 women, the mean (SD) age was 62.5 (4.4) years (range, 53–72 years), and the mean BMI was 21.7 (1.6) kg/m^2^. The groups did not differ with respect to general characteristics. All participants voluntarily signed an informed consent, and the study protocol was approved by the Medical Ethics Committee of West China Hospital, Sichuan University (WCH024).

### Methods

All patients received conventional treatment, including antitussive and expectorant agents, enteral and parenteral nutritional support, oxygen mask, anti‑infection medications, and correction of water and electrolyte imbalance. In the MV group, the patients received conventional treatment and ventilation under an assist‑control mode. If the hypoxia status of a patient improved, they were switched to synchronized intermittent mandatory ventilation under the pressure support mode for continued ventilation. The working mode was mainly the spontaneous / timed (S/T) mode. The initial values of inspiratory and expiratory pressure were 6–8 cm H2O and 0–2 cm H2O, respectively. After 10 to 20 minutes, they were appropriately increased to 14–18 cm H2O and 4–6 cm H2O, respectively. The treatment time and ventilation parameters were adjusted according to disease severity, as well as based on ventilation performance and blood gas indicators. After continuous ventilation lasting for 2 to 3 hours, the patient rested for 1 to 30 minutes. During MV, intermittent subglottic aspiration was performed to prevent ventilator‑associated pneumonia. The patients included in the BAL‑assisted MV group underwent BAL in addition to receiving conventional treatment and MV. Under a direct guidance of bedside fiberoptic bronchoscopy, an endotracheal catheter was placed into the endoscope body, which was extended into the corresponding lung lobe or bronchial opening for secretion absorption, according to computed tomography (CT) and fiberoptic bronchoscopy visualization results. If the secretions were sticky and difficult to suck out, lavage with 10 to 20 ml of warm saline was repeated until their complete removal, with negative pressure not exceeding 200 mm Hg. The lung tissue was then washed with 20 ml of antibacterial drugs. BAL was performed every 3 days until the total amount of accumulated lavage fluid was between 60 ml and 120 ml. Each procedure lasted for 15 to 25 minutes, and the patients were closely monitored for changes in vital signs.

### Observation indicators

General patient data were recorded, including PI control time, MV duration, temperature recovery time (TRT), and the Clinical Pulmonary Infection Score (CPIS).^12^

The lung function of patients before and after treatment was evaluated. The assessed parameters included maximum voluntary ventilation (MVV), total lung capacity (TLC), forced expiratory volume in 1 second (FEV_1_), maximum mid‑expiratory flow (MMEF), lung compliance (Cdyn), airway resistance (Raw), peak inspiratory pressure (PIP), and work of breathing (WOB).

Blood gas analysis was performed before and after treatment to evaluate blood gas indices, including arterial oxygen saturation (SaO_2_), arterial oxygen pressure (PaO_2_), partial pressure of carbon dioxide in the artery (PaCO_2_), pH value, and the ratio of partial pressure of oxygen in arterial blood to the fraction of inspiratory oxygen concentration (PaO_2_/FiO_2_).

A total of 3 ml of fasting venous blood was collected before and after treatment and centrifuged at 1000 rpm for 10 minutes to collect the supernatant. High‑sensitivity Creactive protein (hs‑CRP), procalcitonin (PCT), tumor necrosis factor α (TNF‑α), high‑mobility group box 1 (HMGB1), interleukin 6 (IL‑6), and macrophage inflammatory protein 1α (MIP‑1α) concentrations were measured by enzyme‑linked immunosorbent assay (ELISA). All ELISA kits were purchased from Shanghai Enzyme‑linked Biotechnology Co., LTD. (Shanghai, China), and the measurements were carried out according to the manufacturer’s instructions.

Treatment efficacy was evaluated as follows: a patient was considered cured when the clinical symptoms and pulmonary rales disappeared, and chest CT imaging showed no signs of infection. When the clinical symptoms improved substantially and the chest imaging findings indicated that the lesion was absorbed by at least 50%, treatment was considered obviously effective.

The treatment was deemed effective when the clinical symptoms improved and the lesion was absorbed by 20% to 50%, based on chest imaging data. If the clinical symptoms and chest imaging findings showed no improvement or exacerbation of the infection focus, the treatment was considered ineffective. Clinical effective rate (CER) was calculated using the following formula: CER = (number of cured + number of obvious effective + number of effective) / total number of patients × 100%.

### Statistical analysis

Statistical analysis was performed using SPSS 22.0 (IBM Corp., Armonk, New York, United States). Count and measurement data were presented as frequency or percentage and mean with SD, respectively. Variables were compared with the χ^2^ test and the *t* test, as appropriate. A *P* value below 0.05 was assumed as significant.

## RESULTS

### Ventilation strategy

**FIGURE 1:**
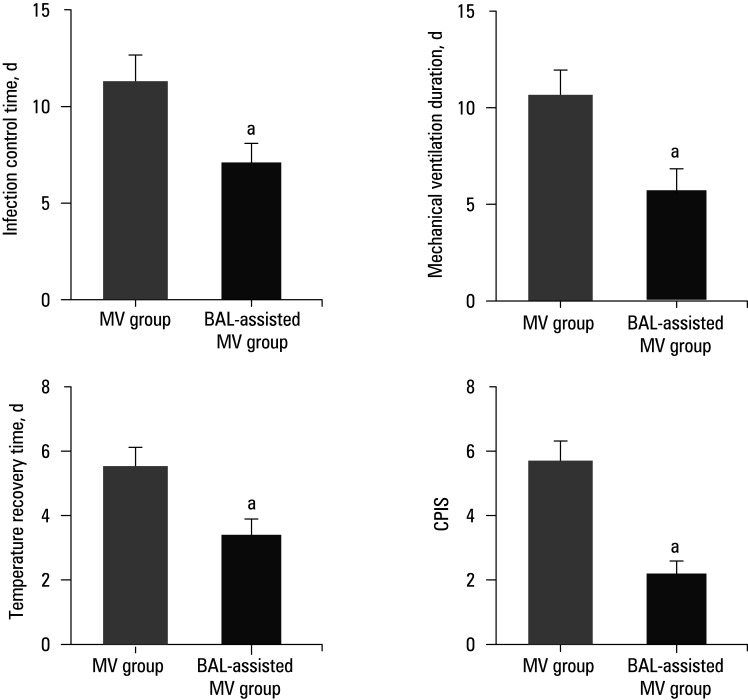
Comparison of treatment efficacy

Differences in treatment efficacy between MV and BAL‑assisted MV are shown in FIGURE 1. The PI control time, MV duration, and TRT in the BAL‑assisted MV group were substantially shorter, and CPIS was lower than in the MV group (*P* <0.05).

a *P* <0.05 vs the MV group

Abbreviations: BAL, bronchoalveolar lavage; CPIS, Clinical Pulmonary Infection Score; MV, mechanical ventilation

**FIGURE 2:**
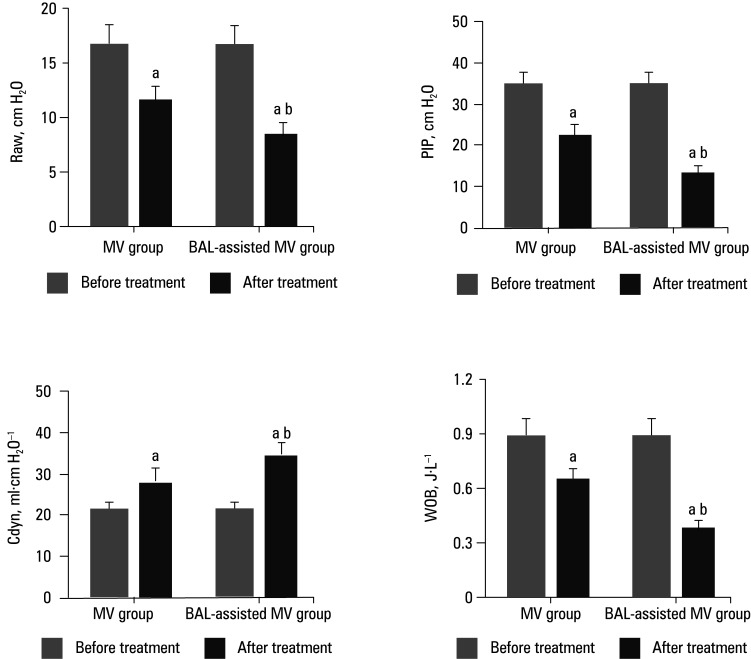
Parameters of respiratory mechanics

Parameters of respiratory mechanics A comparison of parameters reflecting respiratory mechanics before and after treatment is illustrated in FIGURE 2. Post‑treatment Raw, PIP, and WOB values were significantly reduced in both groups, while Cdyn values were increased, as compared with those before treatment. Furthermore, the post‑treatment Raw, PIP, and WOB values in the BAL‑assisted MV group were lower, while the Cdyn value was greater than the values observed in the MV group (all *P* <0.05).

**a** *P* <0.05 vs pretreatment values **b** *P* <0.05 vs the MV group

Abbreviations: Cdyn, lung compliance; PIP, peak airway pressure; Raw, airway resistance; WOB, work of breathing; others, see FIGURE 1

### Parameters of lung function

**FIGURE 3:**
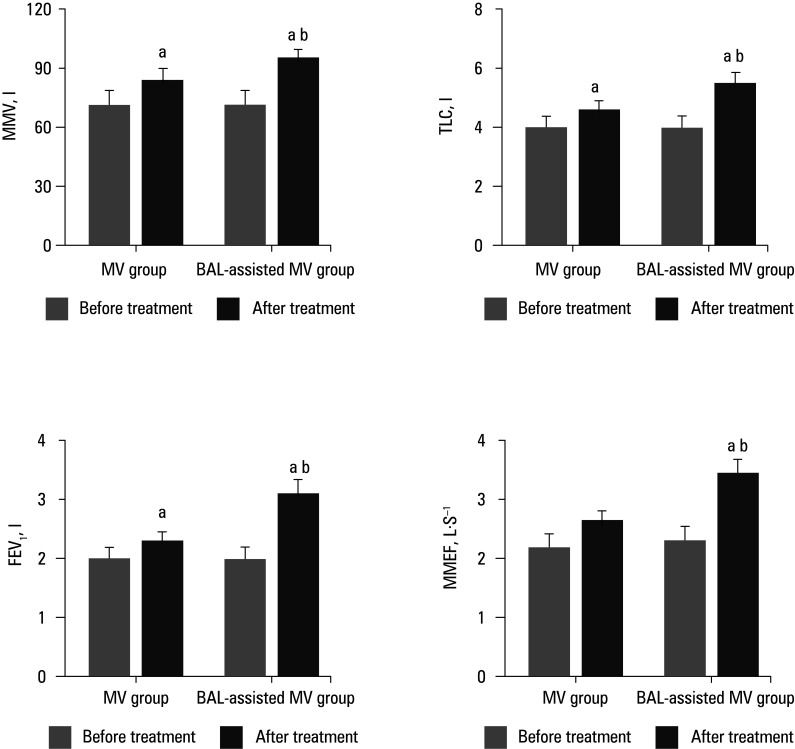
Lung function parameters following different treatments

Lung function parameters before and after treatment were compared in both groups (FIGURE 3). The postoperative values of MMV, TLC, FEV_1 _, and MMEF in all patients were significantly increased, as compared with the preoperative values. Furthermore, the MMV, TLC, FEV_1 _, and MMEF values in the BAL‑assisted MV group exhibited a greater increase than in the MV group (*P* <0.05).

**a** *P* <0.05 vs pretreatment values **b** *P* <0.05 vs the MV group

Abbreviations: FEV_1_ , forced expiratory volume in 1 second; MMEF, maximum mid-expiratory flow; MMV, maximum minute ventilation; TLC, total lung capacity; others, see FIGURE 1

### Parameters of blood gas function

**FIGURE 4:**
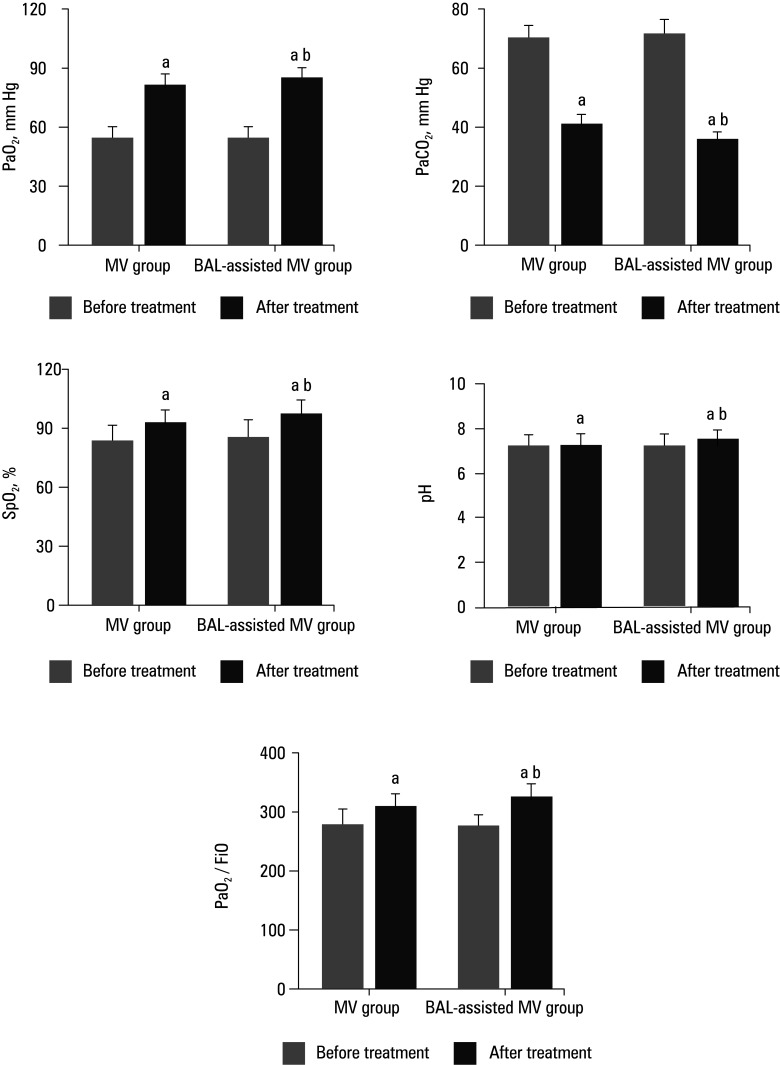
Comparison of blood gas indices before and after treatment

A comparison of the differences in blood gas indices between the MV group and the BAL‑assisted MV group before and after the treatment is illustrated in FIGURE 4. The postoperative PaO_2_, SpO_2_, pH, and PaO_2 _/FiO_2 _levels in both groups were significantly increased, while PaCO_2 _was significantly decreased, as compared with the preoperative levels. The increase in post‑treatment PaO_2_, SpO_2_, pH, and PaO_2_/FiO_2_ levels and the decrease in the PaCO_2 _level in the BAL‑assisted MV group were greater than those observed in the MV group (*P* <0.05).

**a** *P* <0.05 vs pretreatment values **b** *P* <0.05 vs the MV group

Abbreviations: PaO_2_ , arterial oxygen pressure; PaO2_2_ /FiO_2_ , the ratio of partial pressure of oxygen in arterial blood to the fraction of inspiratory oxygen concentration; PaCO_2_, partial pressure of carbon dioxide; SpO_2_, arterial oxygen saturation, others, see FIGURE 1

### Peripheral blood inflammatory markers

**FIGURE 5:**
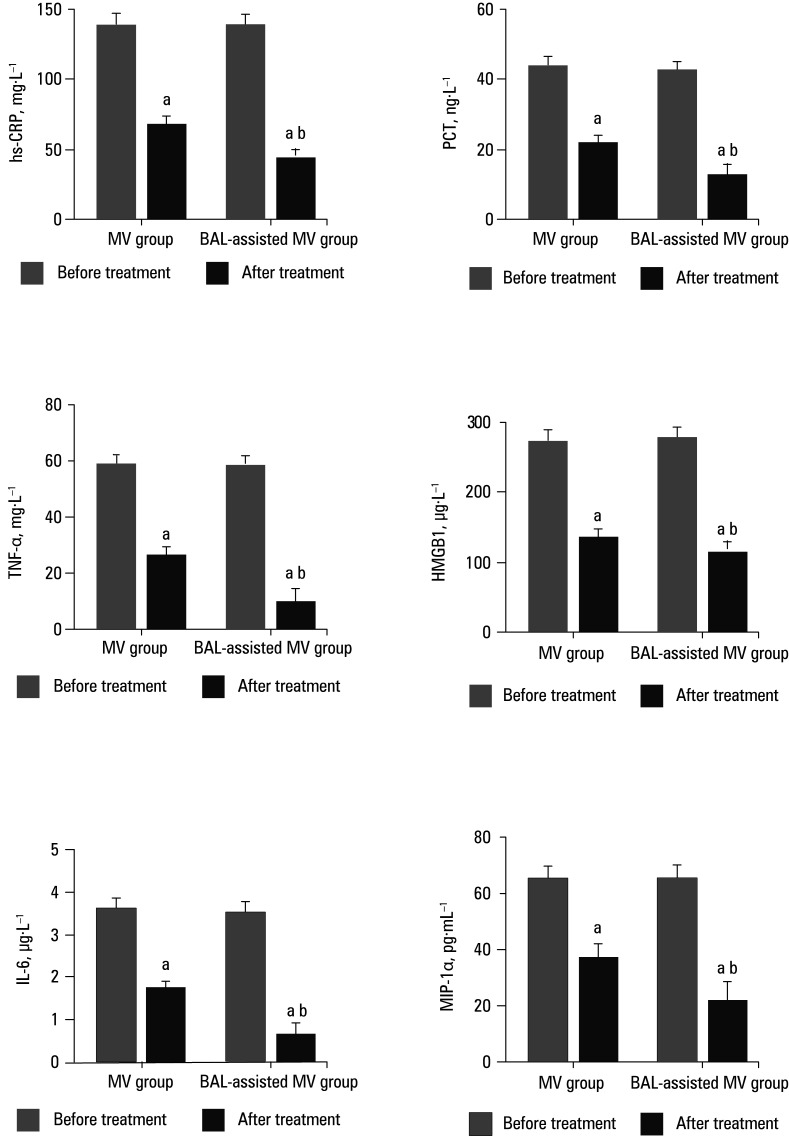
Inflammatory factors in peripheral blood

Differences in the levels of inflammatory parameters are shown in FIGURE 5. Post‑treatment levels of hs‑CRP, PCT, TNF‑α, HMGB1, IL‑6, and MIP‑1α in both groups were significantly reduced, as compared with the pretreatment levels. In comparison with the patients treated with MV alone, the post‑treatment levels of hs‑CRP, PCT, TNF‑α, HMGB1, IL‑6, and MIP‑1α in the BAL‑assisted MV group were significantly lower.

**a** *P* <0.05 vs pretreatment values **b** *P* <0.05 vs the MV group

Abbreviations: hc-CRP, high-sensitivity C-reactive protein; HMGB1, highmobility group box 1; IL-6, interleukin 6; MIP-1a, macrophage inflammatory protein 1α; PCT, procalcitonin; TNF-α, tumor necrosis factor α; others, see FIGURE 1

### Treatment efficacy

**FIGURE 6:**
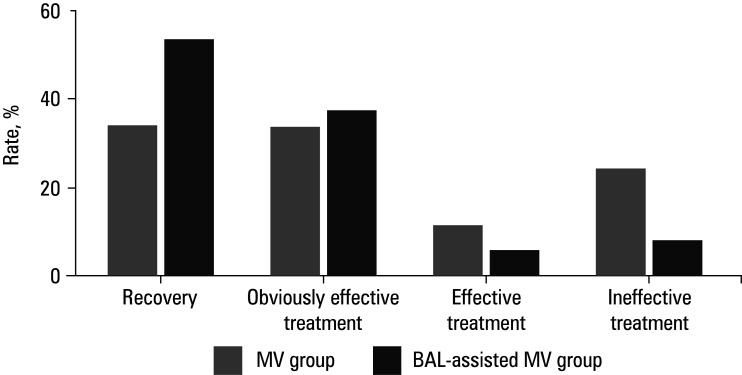
Clinical effective rate following different treatments

Abbreviations: see FIGURE 1

A comparison of clinical outcomes between the MV group and the BAL‑assisted MV group is demonstrated in FIGURE 6. In the MV group, 15 patients were cured (33.33%), 15 were obviously effectively treated (33.33%), 5 were effectively treated (11.11%), and 10 individuals were ineffectively treated (22.22%), yielding a CER of 77.78%. In the BAL‑assisted MV group, 23 patients (51.11%) were cured, 17 were obviously effectively treated (37.78%), 2 were effectively treated (4.44%), and 3 patients were cured (6.67%); thus, the CER reached 93.33%. Overall, the CER among the patients treated with BAL‑assisted MV was higher than in the patients treated with MV alone (*P* <0.05).

## DISCUSSION

The postoperative respiratory function of patients undergoing thoracic surgery can be reduced to varying degrees, and the probability of respiratory insufficiency is increased for a short period of time, as is the risk of complications, such as respiratory failure, PI, and atelectasis. All these factors affect the rehabilitation process.^13^^;^^14^ The course of postoperative PI in patients treated with LC surgery is more complex, and the disease progresses more rapidly than in patients with ordinary pneumonia. This results in a more significant decline in respiratory function and greater difficulty of managing patients requiring assisted MV treatment.^15^^;^^16^ Treatment strategies in LC patients with postoperative PI usually comprise MV combined with routine anti‑infection treatment. However, clinical symptom control is often poor, and drug‑resistant strains are easy to form, which increases the in‑hospital infection rate and prolongs the rehabilitation process of patients.^17^ Moreover, conventional sputum aspiration can cause bronchial mucosal injury and increase the amount of secretions in the lower lobe bronchi.^18^ Therefore, improvement of therapeutic efficacy, respiratory function, and long‑term prognosis of patients who develop PI following LC surgery has become a focus of current research.

BAL‑assisted MV is a treatment method that involves thorough cleaning of the lung segment and the bronchi below under direct vision; thus, it can effectively clear mucus secretions from the lungs.^19^ In addition, direct administration of antibiotics through a fiberscope can significantly improve lung tissue ventilation and air exchange.^20^ BAL‑assisted MV combines airway obstruction removal, local high‑concentration drug therapy, sputum culture, and other functions. Perfusion with normal saline does not cause adverse effects in the lung tissue.^21^ This study compared the effects of fiberoptic BAL‑assisted MV treatment vs MV alone on inflammatory response and respiratory function in post‑LC surgery patients with PI. Postoperative PI exacerbates the systemic inflammatory response and is associated with elevated levels of various proinflammatory indices due to great trauma and stress caused by the LC operation. hs‑CRP is the most sensitive indicator of inflammation; its level is significantly increased in the early stage of inflammatory response, and can reflect the progression and outcome of the disease.^22^ PCT levels increase in the acute stage of inflammation and can be used to evaluate the activity of inflammatory response in the body.^23^ TNF‑α is an important proinflammatory factor whose level increases gradually with the progression of the disease. It can activate neutrophils and lymphocytes, enhance vascular endothelial cell permeability, and promote synthesis and release of other cytokines.^24^ HMGB1 is a receptor for end‑product of advanced glycation program and a member of the toll‑like receptor family. It plays a potential role in mediating late inflammatory response and increases rapidly in the first 24 hours of PI.^25^ IL‑6 is an inflammatory factor that can be used for early diagnosis, disease assessment, treatment efficacy monitoring, and prognosis evaluation in infectious diseases.^26^ In acute and chronic inflammatory responses, MIP‑1α binds to its receptors and induces changes in the functional activity of various target cells through signal transduction, thereby promoting the recruitment of proinflammatory cells to the site of the lesion.^27^ In our study, post‑treatment serum levels of hs‑CRP, PCT, TNF‑α, HMGB1, IL‑6, and MIP‑1α were significantly decreased in both study groups. However, treatment with BAL‑assisted MV resulted in a greater decrease of inflammatory factor levels than MV alone. This shows that BAL‑assisted MV could help locally reduce inflammation by monitoring inflammation in the lung, diluting and cleaning inflammatory secretions, reducing the action of bacterial toxins, and inhibiting synthesis and se‑ cretion of inflammatory factors.

Patients who develop PI after LC surgery experience severe respiratory dysfunction, mainly manifested as dual dysfunction of lung ventilation and air exchange.^28^ We showed that respiratory mechanics (Raw, PIP, and WOB) decreased, and Cdyn increased in individuals with post‑LC surgery PI after both MV and BAL‑assisted MV treatment. Analysis of lung function parameters suggested that post‑treatment MMV, TLC, FEV_1 _, and MMEF values increased in both groups; however, BAL‑assisted MV was associated with a greater improvement in respiratory mechanics and lung function indices than MV alone. BAL‑assisted MV can completely clear sticky secretions generated in the airway of PI patients, maintain airway patency, reduce airway resistance, enhance MV, and thus improve alveolar ventilation and compliance.^29^^;^^30^ Secondly, analysis of blood gas function parameters indicated that PaO_2 _, SpO_2 _, pH, and PaO_2_/FiO_2_ values were increased, while PaCO_2_ was decreased in LC patients with PI after both MV and BAL‑assisted MV treatment; however, the latter method resulted in a greater improvement of blood gas function indices. Reduction of inflammatory parameter levels can quickly eliminate alveolar edema, prevent adhesion, proliferation, and calcification of endothelial cells, fibrin, and epithelial cells of lung tissue, protect alveolar and airway function, and promote lung function rehabilitation.^31^ In addition, BAL‑assisted MV can optimize microcirculation of the body, relieve local hypoxic‑ischemia state and blood circulation at the infection focus, inhibit pathogen activity, improve clinical symptoms, and promote lung function recovery.^32^ In the process of BAL‑assisted MV treatment, local blood circulation function can be quickly restored with antibacterial drugs. Additionally, such treatment reduces the amount of drugs required and prevents low local blood concentration in the focal area.

The CERs in the MV and BAL‑assisted MV groups were 77.78% and 93.33%, respectively. Such results indicate that BAL‑assisted MV is more efficient in eliminating and reducing mediastinal oscillation and improving the respiratory function of patients. At the same time, it can quickly correct hypoxemia, improve tissue oxygenation level, separate atrophied and collapsed alveoli, increase lung gas exchange area, and accelerate gas dispersion.^33^^;^^34^ BAL‑assisted MV allows for accurate suction of secretions in the diseased areas of the lung under direct vision. Diluting secretions with normal saline before aspiration can improve clearance effectiveness and ventilation function of the lungs. However, liquid lavage can stimulate airway and lung mucosa, induce cough, and promote sputum discharge.^35^ By absorbing deep sputum and lavage solution for pathogen culture, the culture results are more accurate, which is conducive to improved guidance and symptomatic treatment.

## CONCLUSIONS

We showed that BAL‑assisted MV treatment can effectively control the respiratory symptoms in post‑LC surgery patients with PI. The improvement of respiratory mechanics, lung function, and blood gas indices was reflective of systemic inflammatory response inhibition. However, to prevent potential treatment complications, close patient monitoring and intensive nursing care during fiberoptic BAL‑assisted MV are indicated. In conclusion, BAL‑assisted MV can be applied in treating LC patients with postoperative PI, and is associated with better treatment outcomes than MV alone.

## References

[BIBR-1] Singh S., Goyal D., Raman K. (2023). RNA profile of immuno‐magnetically enriched lung cancer associated exosomes isolated from clinical samples. Cancer Genet.

[BIBR-2] Samara K.D., Antoniou K.M., Karagiannis K. (2012). Expression profiles of toll‐like receptors in non‐small cell lung cancer and idiopathic pulmonary fibrosis. Int J Oncol.

[BIBR-3] Sigel K.M., Stone K., Wisnivesky J.P. (2019). Short‐term outcomes for lung cancer resection surgery in HIV infection. AIDS.

[BIBR-4] Xin X.L., Ning L.Y., Wei Z.X. (2004). Effects of prone position ventilation on inflammatory factors in blood and bronchial alveolar lavage fluid of acute respiratory distress syndrome dogs caused by pulmonary and extrapulmonary insults [in Chinese. Zhonghua Yi Xue Za Zhi.

[BIBR-5] Zhang J., Jiang M.X., Zheng Y. (2016). Comparison of laparoscopy and open surgery in treating severe acute pancreatitis and its relative aftercare. J Biol Regul Homeost Agents.

[BIBR-6] Evrevin M., Hermet L., Guillet‐Caruba C. (2021). Improving tuberculosis management in prisons: impact of a rapid molecular point‐of‐care test. J In‐ fect.

[BIBR-7] Lin L.Y., Chang M.H., Lee W.J. (2014). Paraneoplastic limbic encephalitis associated with adenocarcinoma of lung. Acta Neurol Taiwan.

[BIBR-8] Darie A.M., Khanna N., Jahn K. (2022). Fast multiplex bacterial PCR of bronchoalveolar lavage for antibiotic stewardship in hospitalised patients with pneumonia at risk of Gram‐negative bacterial infection (Flagship II): a multicentre, randomised controlled trial. Lancet Respir Med.

[BIBR-9] Xu X.H., Fan H.F., Shi T.T. (2021). Influence of the timing of bronchoscopic al‐ veolar lavage on children with adenovirus pneumonia: a comparative study. BMC Pulm Med.

[BIBR-10] Rouze A., Voiriot G., Guivarch E. (2018). Inflammatory cellular response to mechanical ventilation in elastase‐induced experimental emphysema: role of preexisting alveolar macrophages infiltration. Biomed Res Int.

[BIBR-11] Shi Y., Huang Y., Zhang T.T. (2019). Chinese guidelines for the diagnosis and treatment of hospital‐acquired pneumonia and ventilator‐associated pneumonia in adults (2018 Edition. J Thorac Dis.

[BIBR-12] Gunalan A., Sistla S., Sastry A.S. (2021). Concordance between the National Healthcare Safety Network (NHSN) surveillance criteria and Clinical Pulmonary Infection Score (CPIS) criteria for diagnosis of Ventilator‐Associated Pneumonia (VAP. Indian J Crit Care Med.

[BIBR-13] Bassetti M., Eckmann C., Giacobbe D.R. (2020). Post‐operative abdominal infections: epidemiology, operational definitions, and outcomes. Intensive Care Med.

[BIBR-14] Du J., Fu Y., Lv Y. (2022). Preoperative localization for lung nodules: a meta‐analysis of bronchoscopic versus computed tomography guidance. Wideochir Inne Tech Maloinwazyjne.

[BIBR-15] Jo Y., Lee K., Chang Y. (2020). Effects of an alveolar recruitment maneuver during lung protective ventilation on postoperative pulmonary complications in elderly patients undergoing laparoscopy. Clin Interv Aging.

[BIBR-16] Liu H., Dilger J.P., Lin J. (2020). Effects of local anesthetics on cancer cells. Pharmacol Ther.

[BIBR-17] Tamura A., Suzuki J., Fukami T. (2015). Chronic pulmonary aspergillosis as a sequel to lobectomy for lung cancer. Interact Cardiovasc Thorac Surg.

[BIBR-18] Colley N., Mani H., Ninomiya S. (2020). Effective catheter manoeuvre for the removal of phlegm by suctioning: a biomechanical analysis of experts and novices. J Med Biol Eng.

[BIBR-19] Tsang H.F., Yu A.C.S., Jin N. (2022). The clinical application of metagenomic next‐generation sequencing for detecting pathogens in bronchoalveolar lavage fluid: case reports and literature review. Expert Rev Mol Diagn.

[BIBR-20] Patyk I., Rybacki C., Kalicka A. (2019). Simvastatin therapy and bronchoalveolar lavage fluid biomarkers in chronic obstructive pulmonary disease. Adv Exp Med Biol.

[BIBR-21] Tousson E., El‐Gharbawy D.M. (2023). Impact of saussurealappa root extract against copper oxide nanoparticles induced oxidative stress and toxicity in rat cardiac tissues. Environ Toxicol.

[BIBR-22] Pope J.E., Choy E.H. (2021). C‐reactive protein and implications in rheumatoid arthritis and associated comorbidities. Semin Arthritis Rheum.

[BIBR-23] Maleitzke T., Dietrich T., Hildebrandt A. (2023). Inactivation of the gene encoding procalcitonin prevents antibody‐mediated arthritis. Inflamm Res.

[BIBR-24] Zhou Z.D., Yang H.Z., Wang X.Q. (2023). Blocking CCR10 expression activates m6A methylation and alleviates vascular endothelial cell injury. Dis‐ cov Med.

[BIBR-25] Dulmovits B.M., Tang Y., Papoin J. (2022). HMGB1‐mediated restriction of EPO signaling contributes to anemia of inflammation. Blood.

[BIBR-26] Rose‐John S. (2022). Local and systemic effects of interleukin‐6 (IL‐6) in inflammation and cancer. FEBS Lett.

[BIBR-27] Ma D., Ma G.Q. (2020). Mechanism prediction of SimiaoYongan Decoction in treatment of psoriasis arthritis based on network pharmacology [in Chinese. Zhongguo Zhong Yao Za Zhi.

[BIBR-28] Handa Y., Tsutani Y., Mimae T. (2021). Postoperative pulmonary function after complex segmentectomy. Ann Surg Oncol.

[BIBR-29] Di M.E., Yang D., Di Y.P. (2020). Using bronchoalveolar lavage to evaluate changes in pulmonary diseases. Methods Mol Biol.

[BIBR-30] Jin Z., Zhang W., Liu H. (2022). Potential therapeutic application of local anesthetics in cancer treatment. Recent Pat Anticancer Drug Discov.

[BIBR-31] Wu X., Liu Z., Hu L. (2018). Exosomes derived from endothelial progenitor cells ameliorate acute lung injury by transferring miR‐126. Exp Cell Res.

[BIBR-32] Zhao H., Gu H., Liu T. (2018). Analysis of curative effect of adjuvant therapy with bronchoalveolar lavage on COPD patients complicated with pneumonia. Exp Ther Med.

[BIBR-33] Lentz S., Roginski M.A., Montrief T. (2020). Initial emergency department mechanical ventilation strategies for COVID‐19 hypoxemic respiratory failure and ARDS. Am J Emerg Med.

[BIBR-34] Mazzeo A.T., Fanelli V., Mascia L. (2013). Brain‐lung crosstalk in critical care: how protective mechanical ventilation can affect the brain homeostasis. Minerva Anestesiol.

[BIBR-35] Cavarra E., Martorana P.A., Gambelli F. (1996). Neutrophil recruitment into the lungs is associated with increased lung elastase burden, decreased lung elastin, and emphysema in alpha 1 proteinase inhibitor‐deficient mice. Lab Invest.

